# A nomogram for predicting 28-day mortality in elderly patients with acute kidney injury receiving continuous renal replacement therapy: a secondary analysis based on a retrospective cohort study

**DOI:** 10.1186/s12882-024-03628-5

**Published:** 2024-06-11

**Authors:** Xiang Li, Yang Li, Cheng-Juan Fan, Zhan-feng Jiao, Yi-Ming Zhang, Na-na Luo, Xiao-Fen Ma

**Affiliations:** Department of Nephrology, Affiliated Hospital of Jining Medical University, Jining, 271000 China

**Keywords:** Elderly patients, AKI, Continuous renal replacement therapy, Nomogram

## Abstract

**Background:**

Acute kidney injury (AKI) is a common and serious condition, particularly among elderly patients. It is associated with high morbidity and mortality rates, further compounded by the need for continuous renal replacement therapy in severe cases. To improve clinical decision-making and patient management, there is a need for accurate prediction models that can identify patients at a high risk of mortality.

**Methods:**

Data were extracted from the Dryad Digital Repository. Multivariate analysis was performed using least absolute shrinkage and selection operator (LASSO) logistic regression analysis to identify independent risk factors and construct a predictive nomogram for mortality within 28 days after continuous renal replacement therapy in elderly patients with acute kidney injury. The discrimination of the model was evaluated in the validation cohort using the area under the receiver operating characteristic curve (AUC), and calibration was evaluated using a calibration curve. The clinical utility of the model was assessed using decision curve analysis (DCA).

**Results:**

A total of 606 participants were enrolled and randomly divided into two groups: a training cohort (*n* = 424) and a validation cohort (*n* = 182) in a 7:3 proportion. A risk prediction model was developed to identify independent predictors of 28-day mortality in elderly patients with AKI. The predictors included age, systolic blood pressure, creatinine, albumin, phosphorus, age-adjusted Charlson Comorbidity Index (CCI), Acute Physiology and Chronic Health Evaluation II (APACHE II) score, and sequential organ failure assessment (SOFA) score. These predictors were incorporated into a logistic model and presented in a user-friendly nomogram. In the validation cohort, the model demonstrated good predictive performance with an AUC of 0.799. The calibration curve showed that the model was well calibrated. Additionally, DCA revealed significant net benefits of the nomogram for clinical application.

**Conclusion:**

The development of a nomogram for predicting 28-day mortality in elderly patients with AKI receiving continuous renal replacement therapy has the potential to improve prognostic accuracy and assist in clinical decision-making.

**Supplementary Information:**

The online version contains supplementary material available at 10.1186/s12882-024-03628-5.

## Introduction

AKI stands as a pervasive and critical medical concern that is particularly significant in the realm of intensive and critical care medicine for elderly individuals. According to a comprehensive multicenter cross-sectional study, over half of intensive care unit patients have experienced AKI [1]. Given the increasing global elderly population, the vulnerability of elderly individuals to AKI is notably heightened, with a substantial increase in incidence corresponding to age [2, 3]. This heightened susceptibility not only exposes them to adverse outcomes such as mortality or progression to chronic kidney disease (CKD) but also elevates the risk of necessitating prolonged dialysis [4]. Furthermore, reports indicate a notably elevated mortality rate among elderly AKI patients compared to their younger counterparts [5], with an even graver prognosis for those requiring replacement therapy [6, 7].

The increased susceptibility of the elderly to AKI is intricately linked to factors such as diminished renal function, age-related decline in glomerular filtration rate (GFR), alterations in renovascular reactivity, polypharmacy, and the presence of comorbidities [5, 8]. In instances of severe AKI, continuous renal replacement therapy has emerged as a life-saving intervention, contributing to the maintenance of fluid balance and electrolyte stability. However, the application of continuous renal replacement therapy is not devoid of risks, encompassing potential infections, bleeding complications, and mechanical issues, each posing threats to patient well-being [9]. Given the primacy of elderly healthcare, it is imperative to direct urgent attention to this demographic. Consequently, there is a critical need to delve into the exploration and development of predictive models adept at efficiently identifying elderly AKI patients undergoing continuous renal replacement therapy who face an elevated risk of mortality. This proactive identification can pave the way for timely interventions, ultimately enhancing survival rates for this vulnerable population.

## Methods

### Patient population

This research is based on a secondary analysis of a retrospective cohort study [10]. To obtain the necessary data, the researchers accessed the DATADRYAD data repository (https://datadryad.org/stash) using the DOI identifier 10.5061/dryad.6v0j9. The original study collected data from January 2009 to September 2016 at two medical institutions: Yonsei University Health System Severance Hospital and National Health Insurance Service Medical Center Ilsan Hospital, both located in Korea.

The study focused on a total of 2,391 patients who underwent continuous renal replacement therapy in the intensive care unit (ICU). Inclusion criteria for the original study: Patients with AKI according to the Acute Kidney Injury Network (AKIN) criteria [11] who require renal replacement therapy. Exclusion criteria: Age < 18 years; pregnant or lactating women; patients with stage 2 or above chronic kidney disease; patients with a history of dialysis, continuous renal replacement therapy, renal obstruction, or kidney transplantation; patients without renal function data prior to admission to the ICU. This study further excluded patients under the age of 65.

Finally, a total of 606 patients with complete data were included in the secondary analysis. This study was approved by the Yonsei University Health System, Severance Hospital Institutional Review Board and followed the provisions of the Declaration of Helsinki (approval no. 4-2016-1073). Since the research data are anonymous, informed consent from patients is not needed for secondary analysis.

### Study parameters and endpoint

Demographic, clinical, and laboratory data were collected at the commencement of continuous renal replacement therapy. This included the patient’s age, sex, body mass index (BMI), comorbidities, systolic blood pressure (SBP), diastolic blood pressure (DBP), mean arterial pressure (MAP), mechanical ventilation (MV), hemoglobin (HGB), white blood cell count (WBC), C-reactive protein (CRP), creatinine (CREA), blood urea nitrogen (BUN), glomerular filtration rate (GFR), albumin (ALB), hydrogen carbonate (HCO3-), blood potassium (K), and blood phosphorus (P). Additionally, AKIN staging (stage II and stage III), CCI [12], SOFA score [13], and APACHE II score [14]were recorded. In the original study, nephrologists determined the decision to implement continuous renal replacement therapy and its timing [10]. The missing values for each variable were presented in Table S1. Subsequent to data cleaning, the missing variables underwent imputation utilizing the random forest method. The study endpoint was defined as death occurring within 28 days after the initiation of continuous renal replacement therapy.

### Selection of predictive variables and development of the prediction model

To ensure sufficient sample sizes for both training and validating the model and thereby ensure its reliability and generalizability, we randomly divided the patients into training and validation cohorts at a ratio of 7:3. In the training cohort, we conducted multivariate analysis using LASSO logistic regression analysis to identify independent risk factors and develop a predictive nomogram for 28-day mortality after continuous renal replacement therapy in elderly patients with acute AKI. To facilitate the integration of these findings into clinical practice, we developed an interactive web-based dynamic nomogram application using Shiny.

### Validation of the prediction model

We assessed the performance of the nomogram using a receiver operating characteristic (ROC) curve and calibration curve. The AUC was used to determine discriminant ability, with values ranging from 0.5 (no discriminant ability) to 1 (complete discriminant ability). Additionally, we performed a DCA to establish the net benefit threshold of prediction.

### Statistical analysis

For normally distributed data, descriptive statistics were presented as mean ± standard deviation, while for non-normally distributed data, median with interquartile range was reported. Categorical variables were expressed as percentages. Differences between the training and validation sets were assessed using chi-square tests for categorical variables, and t-tests or Wilcoxon rank-sum tests for continuous variables. ROC curves were generated using the pROC package in R software. An AUC value between 0.70 and 0.80 indicates moderate discrimination, while an AUC exceeding 0.80 indicates high discrimination. Model calibration was evaluated through calibration plots generated using the calibrate package in R software. The alignment of the calibration curve with a reference line reflects the effectiveness of model calibration. The Brier score was calculated to assess model predictive performance. Ranging from 0 to 1, the Brier score is a common metric for measuring the accuracy of probability predictions, with lower values indicating higher predictive accuracy. Further analysis involved calculating intercept and slope on the calibration curve to explore the relationship between dependent and independent variables. The intercept represents the predicted value of the dependent variable when the independent variable is zero, while the slope represents the rate of change of the dependent variable with respect to changes in the independent variable. Statistical significance was defined as a *p*-value less than 0.05. All analyses were conducted using R software (version 4.2.2) in conjunction with MSTATA software.

## Results

### Baseline characteristics

A total of 606 patients were included in this study, with 360 males and 246 females. The age range of the participants was 65 to 96, with an average age of 74.1. The training cohort consisted of 424 cases, with 246 males and 178 females. The average age of this group was 73.9. The validation cohort included 182 patients, with 114 males and 68 females. The average age of this group was 74.6. There were no significant differences in various indices between the two groups (*P* > 0.05) (Table 1).

**Table 1:** Baseline characteristics of patients in training and validation cohorts

Variables	Total (*n* = 606)	Training Cohorts (*n* = 424)	Validation Cohorts (*n* = 182)	*P*
Age(years)	74.1 ± 5.9	73.9 ± 5.8	74.6 ± 6.0	0.223
Male (%)	360 (59.4)	246 (58)	114 (62.6)	0.289
Myocardial infarction (%)	85 (14.0)	60 (14.2)	25 (13.7)	0.893
Heart failure (%)	124 (20.5)	91 (21.5)	33 (18.1)	0.352
Cerebrovascular disease (%)	80 (13.2)	50 (11.8)	30 (16.5)	0.118
Peripheral vascular disease (%)	35 (5.8)	24 (5.7)	11 (6)	0.853
dementia, *n* (%)	39 (6.4)	29 (6.8)	10 (5.5)	0.536
Diabetes mellitus (%)	259 (42.7)	179 (42.2)	80 (44)	0.692
Hypertension (%)	407 (67.2)	289 (68.2)	118 (64.8)	0.424
COPD (%)	56 (9.2)	35 (8.3)	21 (11.5)	0.201
Potassium (mEq/L)	4.7 ± 1.0	4.7 ± 1.0	4.6 ± 1.1	0.308
Bicarbonate (mEq/L)	16.9 ± 5.2	16.7 ± 5.3	17.6 ± 5.0	0.042
Phosphate (mg/dL)	5.5 ± 2.1	5.6 ± 2.1	5.3 ± 2.0	0.073
BMI (kg/m2)	23.5 ± 4.4	23.5 ± 4.5	23.4 ± 4.1	0.917
SBP (mmHg)	112.7 ± 21.6	113.1 ± 22.0	111.7 ± 20.6	0.477
DBP (mmHg)	57.8 ± 13.6	57.6 ± 13.3	58.2 ± 14.4	0.622
MAP (mmHg)	76.2 ± 14.0	76.4 ± 14.0	75.7 ± 13.9	0.598
MV (%)	460 (75.9)	320 (75.5)	140 (76.9)	0.702
Hemoglobin (g/dL)	9.6 ± 2.1	9.6 ± 2.2	9.6 ± 2.0	0.987
Albumin (g/dL)	2.6 ± 0.6	2.6 ± 0.6	2.6 ± 0.6	0.905
APACHE II score	28.3 ± 7.9	28.3 ± 8.0	28.1 ± 7.8	0.794
SOFA score	11.8 ± 3.5	11.6 ± 3.4	12.0 ± 3.5	0.186
AKI cause, (%)				0.501
Sepsis	418 (69.0)	291 (68.6)	127 (69.8)	
Nephrotoxin	22 (3.6)	19 (4.5)	3 (1.6)	
Ischemia	57 (9.4)	39 (9.2)	18 (9.9)	
Surgery	53 (8.7)	35 (8.3)	18 (9.9)	
Others	56 (9.2)	40 (9.4)	16 (8.8)	
White blood cell (µL)	12250.0 (7362.5, 19045.0)	12445.0 (7847.5, 19160.0)	11690.0 (6957.5, 18812.5)	0.735
BUN (mg/dL)	52.0 (35.0, 74.0)	53.0 (35.0, 75.2)	48.5 (33.2, 71.0)	0.245
Creatinine (mg/dL)	2.4 (1.7, 3.5)	2.4 (1.7, 3.5)	2.3 (1.7, 3.4)	0.912
CRP (mg/L)	100.1 (39.3, 147.9)	100.0 (37.7, 149.4)	101.4 (45.6, 147.6)	0.605
GFR (ml/min)	24.3 (15.4, 35.9)	24.8 (15.0, 35.6)	23.6 (16.8, 36.3)	0.856
CCI	3.0 (2.0, 4.0)	3.0 (2.0, 4.0)	3.0 (2.0, 4.8)	0.716

Data are expressed as mean ± standard deviations, median (interquartile range), or number (%)

Chronic obstructive pulmonary disease, COPD; Charlson comorbidity index, CCI; Body mass index, BMI; Acute kidney injury criteria, AKIN; estimated glomerular filtration rate, GFR; Sequential Organ Failure Assessment score, SOFA score; Acute Physiology and Chronic Health Evaluation II score, APACHE II score; Systolic blood pressure, SBP; Diastolic blood pressure, DBP; Mean arterial pressure, MAP; blood urea nitrogen, BUN; C-reactive protein, CRP

This study compared the all-cause mortality rates in patients with AKI between a test group and a validation group. The mortality rates were determined over a period of 28 days. In the test group, 267 cases of all-cause death occurred within 28 days, resulting in a mortality rate of 63%. Similarly, in the validation group, 112 cases of all-cause death occurred within 28 days, resulting in a mortality rate of 61.5%. Statistical analysis revealed that there was no significant difference in all-cause mortality between the two groups (*p* > 0.05).

This study compared the all-cause mortality rates in patients with AKI between a training cohort and a validation cohort. The mortality rates were determined over a period of 28 days. In the training cohort, 267 cases of all-cause death occurred within 28 days, resulting in a mortality rate of 63%. Similarly, in the validation cohort, 112 cases of all-cause death occurred within 28 days, resulting in a mortality rate of 61.5%. Statistical analysis revealed that there was no significant difference in all-cause mortality between the two cohorts (*P* > 0.05).

### Prediction model construction

**Fig. 1:**
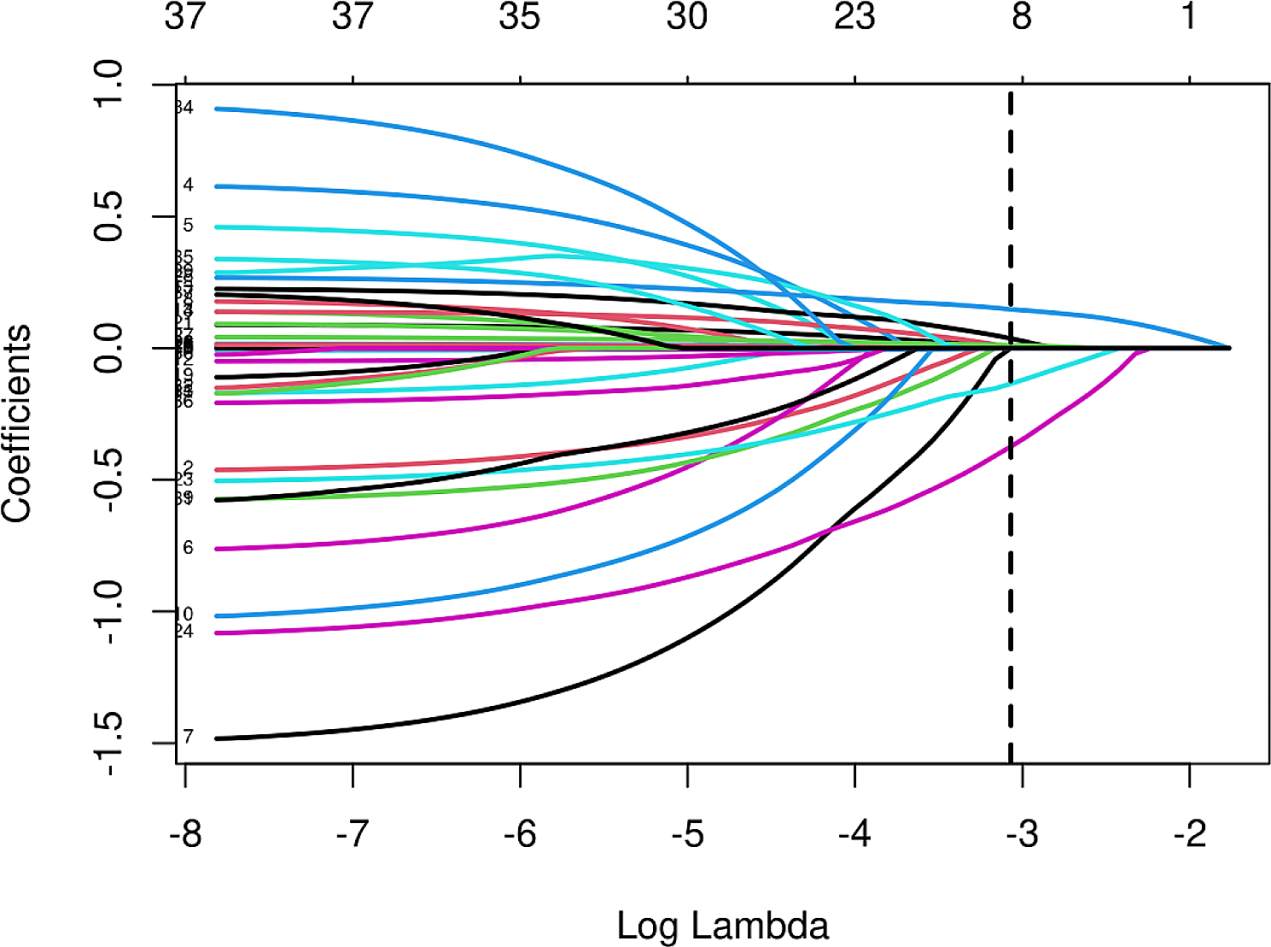
Lasso regression coefficient path plot

**Fig. 2:**
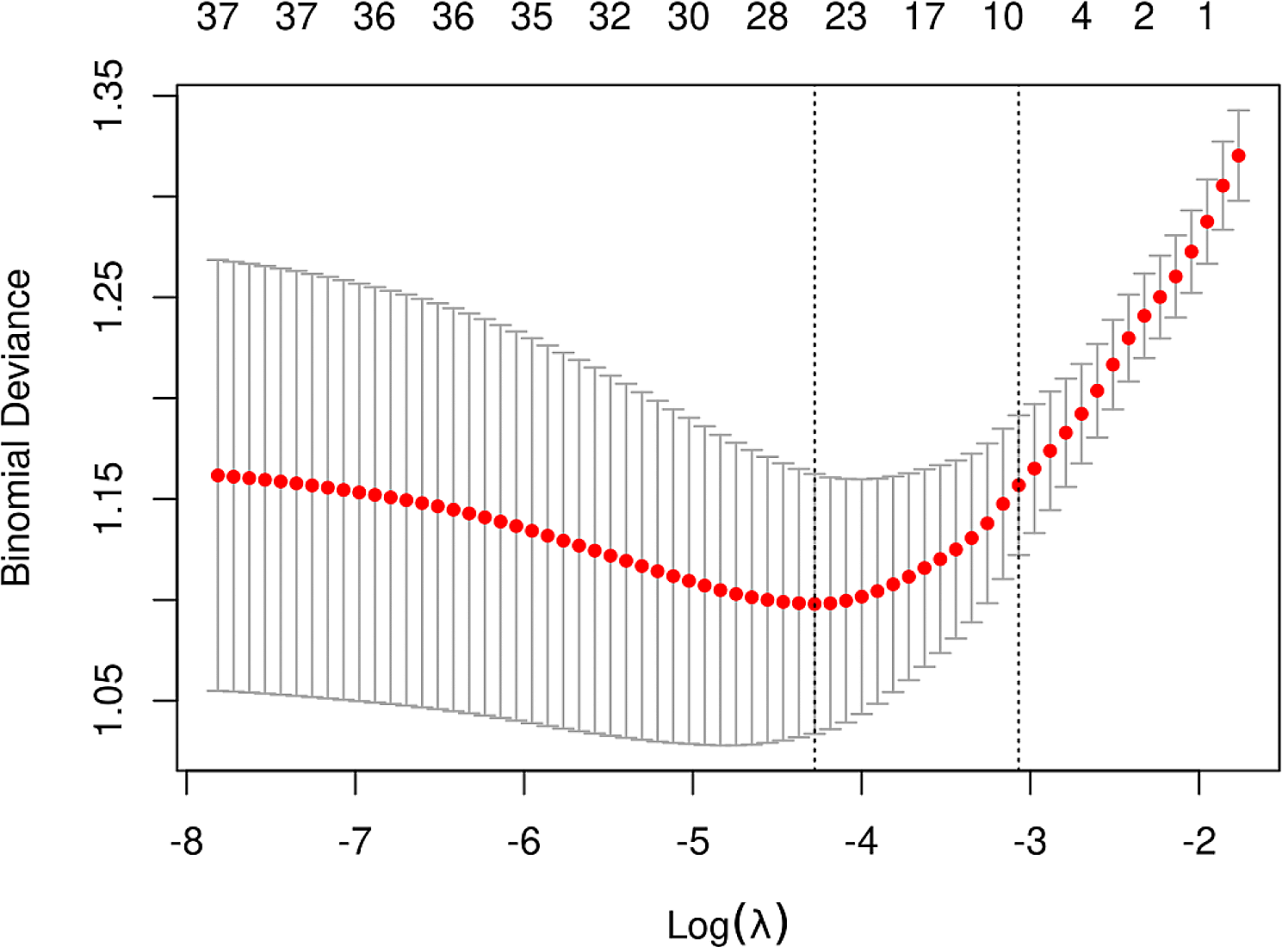
Lasso regression cross-validation plot

**Fig. 3:**
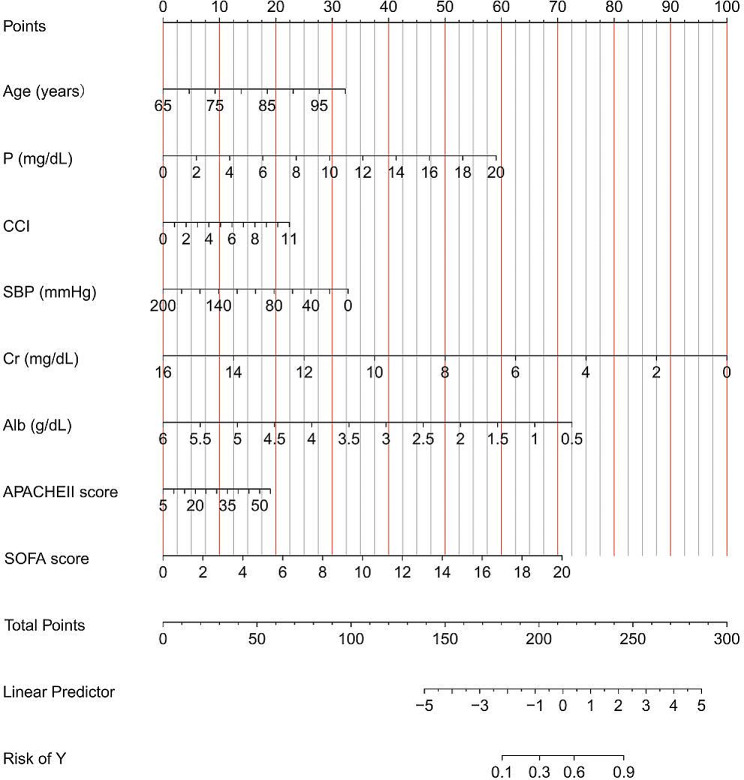
Nomogram prediction model systolic blood pressure, SBP; creatinine, Cr; albumin, Alb; phosphorus, P; Acute Physiology and Chronic Health Evaluation II score, APACHE II score; sequential organ failure assessment score, SOFA score

The initial model encompassed a large number of potential predictors, including age, sex, myocardial infarction, cerebrovascular disease, peripheral vascular disease, dementia, diabetes mellitus (DM), heart failure, hypertension, chronic obstructive pulmonary disease, K, HCO3-, P, BMI, SBP, DBP, MAP, mechanical ventilation, WBC, Hb, BUN, Cr, Alb, CRP, GFR, AKIN staging, CCI, APACHE II score, SOFA score, and cause of acute kidney injury (AKI cause). Following a comprehensive LASSO regression analysis on the training cohort, the number of potential predictors was judiciously narrowed down to eight. The table below delineates the coefficients for these eight predictors (Table 2), with their profiles visually represented in the accompanying figure (Fig. 1). The figure also incorporates a cross-validated error plot of the LASSO regression model (Fig. 2). Embracing a commitment to regularization and parsimony, the final model comprised these eight variables, with the cross-validated error falling within one standard error of the minimum. Subsequent multivariate logistic analyses were executed on distinct cohorts, and the outcomes are meticulously detailed in the subsequent table (Table 3). The ultimate logistic model, featuring 8 independent predictors (age, P, CCI, SBP, Cr, Alb, APACHE II score, and SOFA score), was crafted into a user-friendly nomogram, as elucidated in the ensuing figure (Fig. 3).

**Table 2:** The coefficients of lasso regression analysis

variable	Coefficient
(Intercept)	-0.147235010893
Age	0.000006554494
*P*	0.036363980712
CCI	0.003938178374
SBP	-0.001463657402
Cr	-0.135309382158
Alb	-0.373916256461
APACHE II score	0.010743890509
SOFA score	0.146920292682

systolic blood pressure, SBP; creatinine, Cr; albumin, Alb; phosphorus, P; Acute Physiology and Chronic Health Evaluation II score, APACHE II score; sequential organ failure assessment score, SOFA score

**Table 3:** Results of multivariate logistic regression for training and validation cohorts

Characteristic	Training Cohort				Validation cohort			
	Event *N*	OR^1^	95% CI^1^	*p* value	Event *N*	OR^1^	95% CI^1^	*p* value
Age	267	1.06	1.02, 1.11	0.003	112	1.04	0.98, 1.10	0.207
*P*	267	1.22	1.08, 1.39	0.002	112	1.28	1.05, 1.58	0.018
CCI	267	1.15	1.02, 1.29	0.020	112	1.30	1.09, 1.58	0.004
SBP	267	0.99	0.98, 1.00	0.042	112	0.98	0.97, 1.00	0.053
Cr	267	0.65	0.55, 0.78	< 0.001	112	0.61	0.44, 0.81	0.001
Alb_0h	267	0.41	0.26, 0.63	< 0.001	112	0.65	0.34, 1.19	0.173
APACHE II score	267	1.03	0.99, 1.06	0.120	112	1.00	0.95, 1.05	0.955
SOFA score	267	1.27	1.17, 1.39	< 0.001	112	1.22	1.09, 1.39	0.001

Odds Ratio, OR; Confidence Interval, CI; systolic blood pressure, SBP; creatinine, Cr; albumin, Alb; phosphorus, P; Acute Physiology and Chronic Health Evaluation II score, APACHE II score; sequential organ failure assessment score, SOFA score

### Model performance

**Fig. 4:**
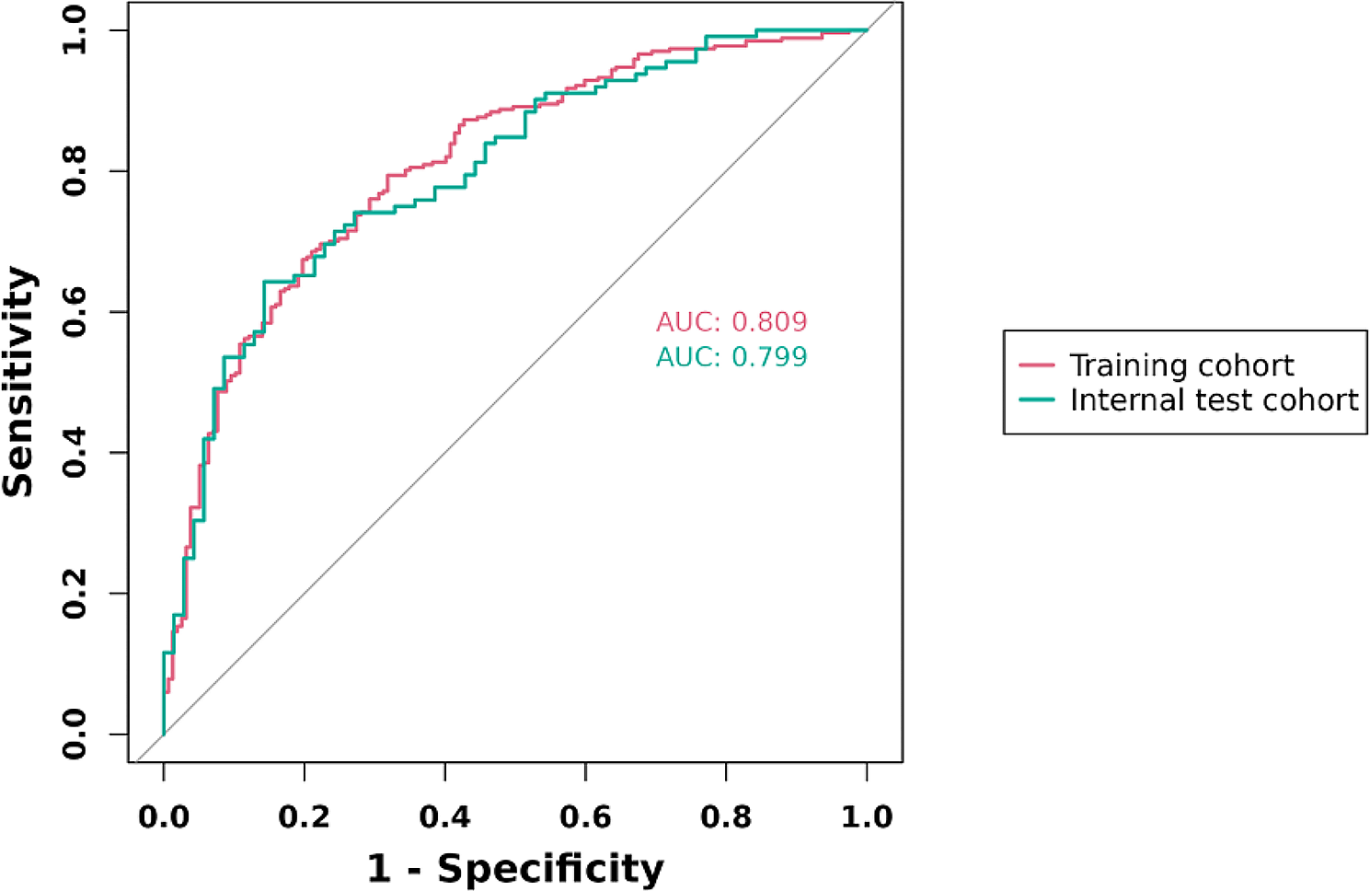
ROC curves of the nomogram prediction model

**Fig. 5:**
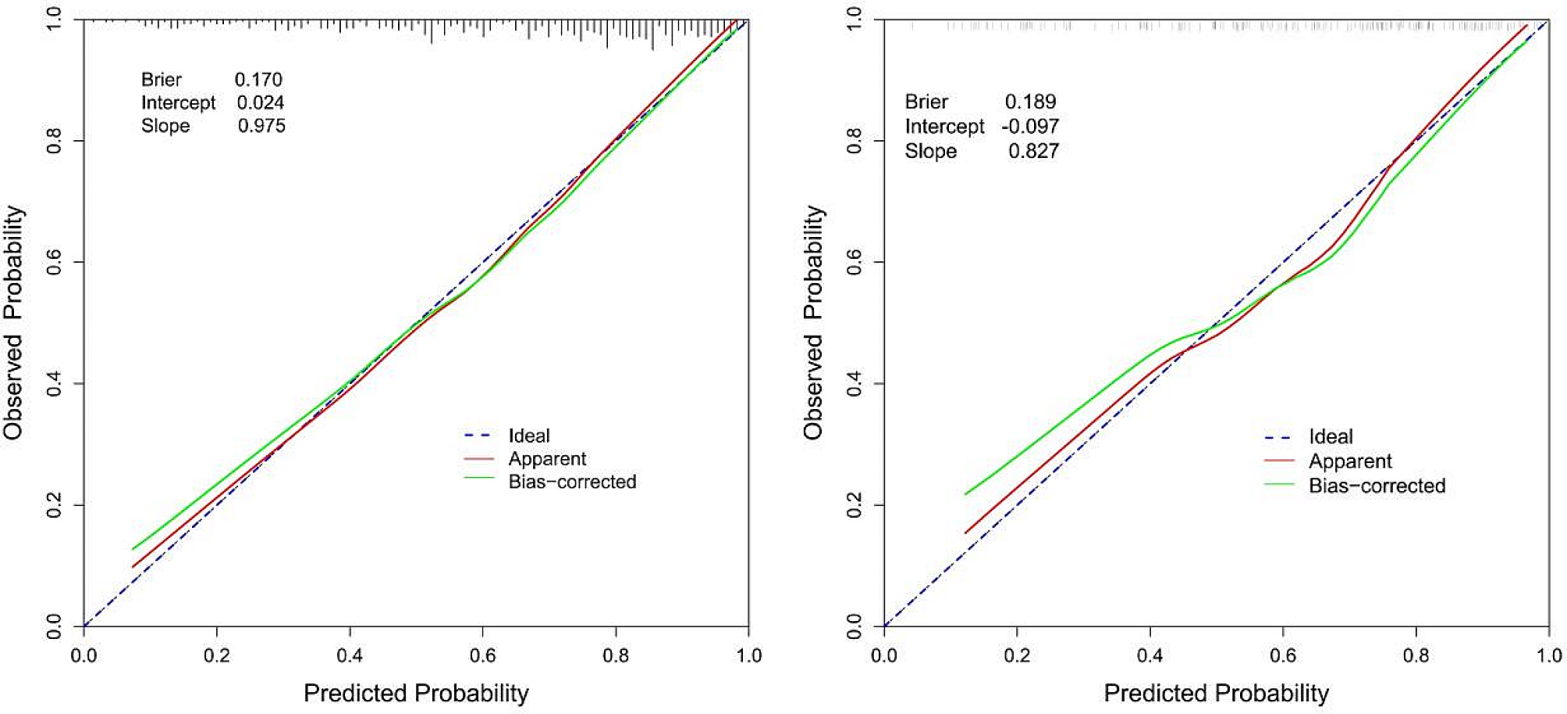
Calibration curve of the nomogram prediction model for the training and validation cohorts

**Fig. 6:**
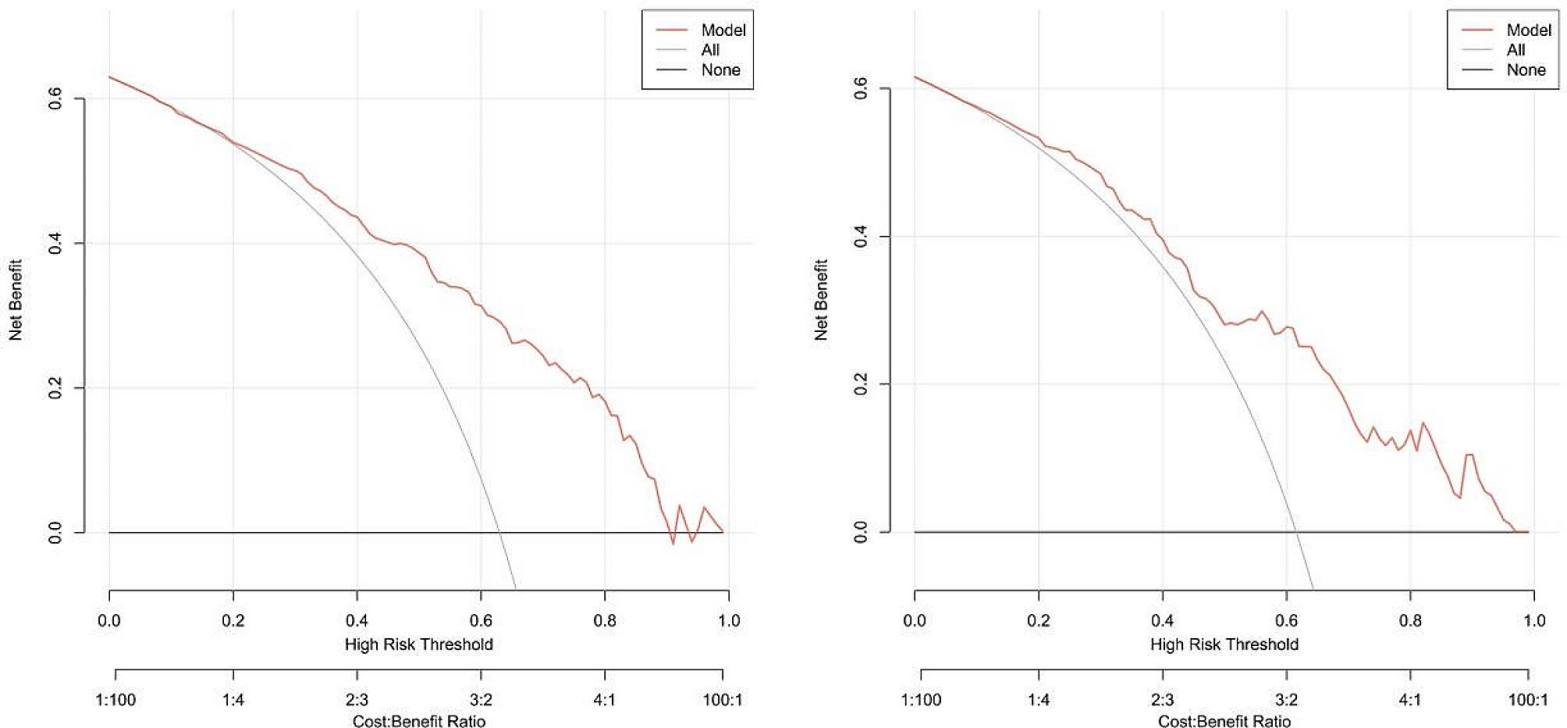
DCA of the nomogram of the training and validation cohorts

The following figures illustrate the AUCs of the model in different cohorts. The AUC for the training cohort was 0.809, as shown in Fig. 4, indicating excellent predictive performance. Additionally, the model exhibited a sensitivity of 0.76, a specificity of 0.71, and an accuracy of 0.74, representing its overall correctness in classification. Similarly, in the validation cohort, the AUC was 0.799, as depicted in Fig. 4, indicating good predictive performance. The calibration curves for the training cohorts (Fig. 5) exhibited an intercept of 0.024 and a slope of 0.975, signifying strong calibration performance of the model. Furthermore, a Brier value of 0.17 was noted, indicating minimal mean squared error between the model’s probability predictions and the actual observed values. These findings underscore the reliability and stability of the model in predictive tasks. Similarly, consistent results were observed in the validation cohorts, further validating the model’s performance. As depicted in Fig. 6, the DCA showcased the nomogram’s superior overall net benefit across a wide and practical range of threshold probabilities, suggesting a high potential for clinical utility.

## Discussion

The retrospective investigation into the 28-day mortality of elderly AKI patients undergoing continuous renal replacement therapy stands as a noteworthy contribution to the fields of nephrology and critical care. The creation and validation of a nomogram for mortality prediction within this demographic provide clinicians with a valuable tool for prognostic assessments. With a substantial cohort of 606 patients, this study ensures a robust sample size, fostering the development of a nomogram grounded in diversity and representation. The focused inclusion of elderly AKI patients receiving continuous renal replacement therapy is particularly pertinent, considering the potential distinct characteristics and treatment nuances within this specific subgroup compared to their younger counterparts.

Although significant progress has been made in the treatment of AKI over the past few decades, the overall mortality rate of AKI patients is still approximately 50%, with critically ill patients reaching up to 80% [15–17]. Mataloun et al.‘s studies on ICU elderly AKI patients have shown that the mortality rate is approximately 63.5%, with a peak of 76.2% [18]. In this study, the inclusion of patients resulted in a mortality rate of over 60%, similar to the results of the aforementioned study. The short-term prognosis of AKI in elderly patients remains bleak due to the severity and complexity of these patients [5].

The selection of age, P, CCI, SBP, Cr, Alb, APACHE II score, and SOFA score as predictors in the nomogram is supported by their statistical significance in the LASSO logistic regression analysis. There are differences in the risk factors for AKI mortality in various studies [19–21], which may be attributed to variations in the application of multivariable regression analysis methods in each study. The application of LASSO logistic regression is methodologically robust, introducing a penalty on regression coefficients to identify a concise set of predictors while mitigating multicollinearity. This methodology enhances interpretability and generalizability, particularly in scenarios characterized by a high-dimensional feature space. Encompassing demographic, clinical, and laboratory variables, these predictors allow for a holistic evaluation of the patient’s overall health and severity of illness, acknowledging the multifaceted contributors to mortality in elderly AKI patients on continuous renal replacement therapy. We chose to include both the SOFA and APACHE II scoring systems with the aim of improving the accuracy of mortality predictions for critically ill patients [22] and gaining a comprehensive understanding of their clinical condition, thereby enhancing the reliability of our research findings.

In this study, the validation cohort’s results affirm the nomogram’s robust performance. With an AUC of 0.799, the nomogram exhibits high discriminative ability. The calibration curve further bolsters the model’s reliability by demonstrating alignment with observed outcomes. These validation metrics underscore the generalizability and suitability of the nomogram across diverse patient populations, reinforcing its potential as a valuable prognostic tool in varied clinical settings. The user-friendly design, as illustrated in Fig. 3, facilitates seamless integration into clinical workflows. Incorporating easily accessible variables enhances the nomogram’s utility for healthcare professionals. The DCA results, indicating significant net benefits for clinical application, emphasize the practical advantages of utilizing the nomogram in guiding clinical decisions. Clinicians can leverage this tool to tailor treatment strategies and intensify monitoring for identified high-risk patients, potentially leading to more timely interventions and improved outcomes.

Despite promising results, acknowledging certain limitations is crucial. External validation in diverse populations and settings will fortify the generalizability of the nomogram. Additionally, ongoing updates and refinements to the model may be necessary as new data emerge. Future research endeavors should explore the integration of emerging biomarkers or dynamic variables to further enhance predictive accuracy.

## Conclusion

The nomogram developed in this study has the capability to predict the 28-day mortality risk in elderly AKI patients undergoing continuous renal replacement therapy. Further validation and integration into routine clinical practice hold promise for improving patient outcomes and guiding resource allocation in a more targeted and informed manner.

### Electronic supplementary material

Below is the link to the electronic supplementary material.

Supplementary Material 1

## Data Availability

The data can be obtained by accessing the DATADRYAD data repository (https://datadryad.org/stash) using the DOI identifier 10.5061/dryad.6v0j9.

## Abbreviations

AKI: Acute kidney injury
LASSO: Least absolute shrinkage and selection operator
AUC: Area under the receiver operating characteristic curve
DCA: Decision curve analysis
CCI: Charlson Comorbidity Index
CKD: Chronic kidney disease
AKIN: Acute Kidney Injury Network
BMI: Body mass index
SBP: Comorbidities, systolic blood pressure
DBP: Diastolic blood pressure
MAP: Mean arterial pressure
MV: Mechanical ventilation
HGB: Hemoglobin
WBC: White blood cell count
CRP: C-reactive protein
CREA: Creatinine
BUN: Blood urea nitrogen
GFR: Glomerular filtration rate
ALB: Albumin
HCO3-: Hydrogen carbonate
K: Blood potassium
P: Blood phosphorus
ROC: Receiver operating characteristic
AKIcause: Cause of acute kidney injury
