# Supplementary material for: A nomogram for predicting 28-day mortality in elderly patients with acute kidney injury receiving continuous renal replacement therapy: a secondary analysis based on a retrospective cohort study
Source: BMC Nephrol. 2024 Jun 11;25:195. doi: 10.1186/s12882-024-03628-5 (PMC11167911; doi:10.1186/s12882-024-03628-5)
Supplement: Supplementary file 1 — Supplementary Material 1 [file 12882_2024_3628_MOESM1_ESM.docx]

| Table S1 A comprehensive report on missing values for each variable | | | |
| --- | --- | --- | --- |
|  | Variable | Miss.freq | Miss.percentage% |
| 1 | Age(years) | 0 | 0 |
| 2 | Male (%) | 0 | 0 |
| 3 | Myocardial infarction (%) | 0 | 0 |
| 4 | Heart failure (%) | 0 | 0 |
| 5 | Cerebrovascular disease (%) | 0 | 0 |
| 6 | Peripheral vascular disease (%) | 0 | 0 |
| 7 | dementia, n (%) | 0 | 0 |
| 8 | Diabetes mellitus (%) | 0 | 0 |
| 9 | Hypertension (%) | 0 | 0 |
| 10 | COPD (%) | 0 | 0 |
| 11 | Potassium (mEq/L) | 7 | 1.1551 |
| 12 | Bicarbonate (mEq/L) | 65 | 10.7261 |
| 13 | Phosphate (mg/dL) | 50 | 8.2508 |
| 14 | BMI (kg/m2) | 11 | 1.8152 |
| 15 | SBP (mmHg) | 0 | 0 |
| 16 | DBP (mmHg) | 0 | 0 |
| 17 | MAP (mmHg) | 0 | 0 |
| 18 | MV (%) | 0 | 0 |
| 19 | Hemoglobin (g/dL) | 2 | 0.33 |
| 20 | Albumin (g/dL) | 4 | 0.6601 |
| 21 | APACHE II score | 10 | 1.6502 |
| 22 | SOFA score | 1 | 0.165 |
| 23 | AKIN Stages | 0 | 0 |
| 24 | AKI cause, (%) | 0 | 0 |
| 25 | White blood cell (μL) | 4 | 0.6601 |
| 26 | BUN (mg/dL) | 2 | 0.33 |
| 27 | Creatinine (mg/dL) | 2 | 0.33 |
| 28 | CRP (mg/L) | 140 | 23.1023 |
| 29 | GFR (ml/min) | 4 | 0.6601 |
| 30 | CCI | 0 | 0 |
